# The role of long non-coding RNAs in genome formatting and expression

**DOI:** 10.3389/fgene.2015.00165

**Published:** 2015-04-29

**Authors:** Pierre-Olivier Angrand, Constance Vennin, Xuefen Le Bourhis, Eric Adriaenssens

**Affiliations:** Cell Plasticity and Cancer – Inserm U908, University of LilleLille, France

**Keywords:** lncRNAs, H19, chromatin organization, transcriptional regulation, post-transcriptional control

## Abstract

Long non-coding RNAs (lncRNAs) are transcripts without protein-coding potential but having a pivotal role in numerous biological functions. Long non-coding RNAs act as regulators at different levels of gene expression including chromatin organization, transcriptional regulation, and post-transcriptional control. Misregulation of lncRNAs expression has been found to be associated to cancer and other human disorders. Here, we review the different types of lncRNAs, their mechanisms of action on genome formatting and expression and emphasized on the multifaceted action of the H19 lncRNA.

The advent of DNA tilling arrays and deep sequencing technologies has revealed that a much larger part of the genome is transcribed into RNAs than previously assumed. It is estimated that up to 70% of the genome is transcribed but only 2% of the human genome codes for proteins (Bertone et al., 2004; Birney et al., 2007; Kapranov et al., 2007; ENCODE Project Consortium, 2012) and RNAs without coding potential are collectively referred as non-coding RNAs (ncRNAs).

Non-coding RNAs include the well-known ribosomal (r) RNAs, ribozymes, transfer (t) RNAs, small nuclear (sn) RNAs, telomere-associated RNAs (TERRA, TERC), as well as a plethora of far less characterized RNAs. Based on their size, these ncRNAs are subdivided into two groups: small ncRNAs (<200 nt) and long ncRNAs [lncRNA (>200 nt)]. Small ncRNAs, such as microRNAs (miRs), small interfering RNAs (siRNAs), or PIWI-interacting RNAs (piRNAs) received much attention and were shown to mainly act as negative regulators of gene expression. In contrast, lncRNAs represent a more functionally diverse class of transcripts. LncRNAs are found in a large diversity of animals species (Guttman et al., 2009; Jia et al., 2010; Pauli et al., 2012), but also in plants (Swiezewski et al., 2009), yeast (Houseley et al., 2008), and even in prokaryotes (Bernstein et al., 1993) and viruses (Reeves et al., 2007). LncRNAs remains poorly conserved among species (Pang et al., 2006; Derrien et al., 2012). However, accumulating evidences indicate that this RNA class plays an important role in a variety of biological processes and may be involved in cancer and other human diseases (Wapinski and Chang, 2011; Tano and Akimitsu, 2012).

Majority of lncRNAs are 5′ capped, 3′ polyadenylated, multi-exonic and are subjected to transcriptional regulation as coding mRNAs (Carninci et al., 2005; Guttman et al., 2010; Cabili et al., 2011; Derrien et al., 2012). Some of the lncRNAs such as XIST, MALAT1, or NEAT1 are almost exclusively localized in the nucleus (Brown et al., 1992; Hutchinson et al., 2007), whereas others are mostly found in the cytoplasm (Coccia et al., 1992; Yoon et al., 2012). In term of genomic organization, lncRNAs can be classified according to their proximity to protein coding genes into five categories: sense, when overlapping one or more exons of another transcript; antisense, when overlapping one or more exons of another transcript on the opposite strand; bidirectional, when its expression and the expression of the neighboring coding transcript on the opposite strand are initiated in close proximity; intronic, when raising from an intron of another transcript; or intergenic, when produced from an independent transcription unit in the interval between two protein coding genes. This crude classification illustrates that lncRNA expression may be controlled by different molecular mechanisms, but it does account neither for their modes of action nor for their cellular functions.

While only a limited number of lncRNAs has been studied, numerous evidences indicate that lncRNAs interact with a plethora of proteins. Furthermore, homologous Watson–Crick base pairing provides an efficient way by which lncRNAs may selectively interact with other nucleic acid species. It is believed that lncRNAs are involved in a diversity of cellular functions through gene expression regulation at different levels including chromatin organization, transcriptional regulation, and post-transcriptional mRNA processing (Mercer et al., 2009; Wilusz et al., 2009).

To complicate matters further, Anderson et al. (2015) recently described that a conserved micropeptide is encoded by a skeletal muscle-specific RNA previously annotated as a putative long non-coding RNA. This finding leads to the proposal that several lncRNAs could also have a biological function through the production of micropeptides.

## LncRNAs in the Control of mRNA Processing

**FIGURE 1:**
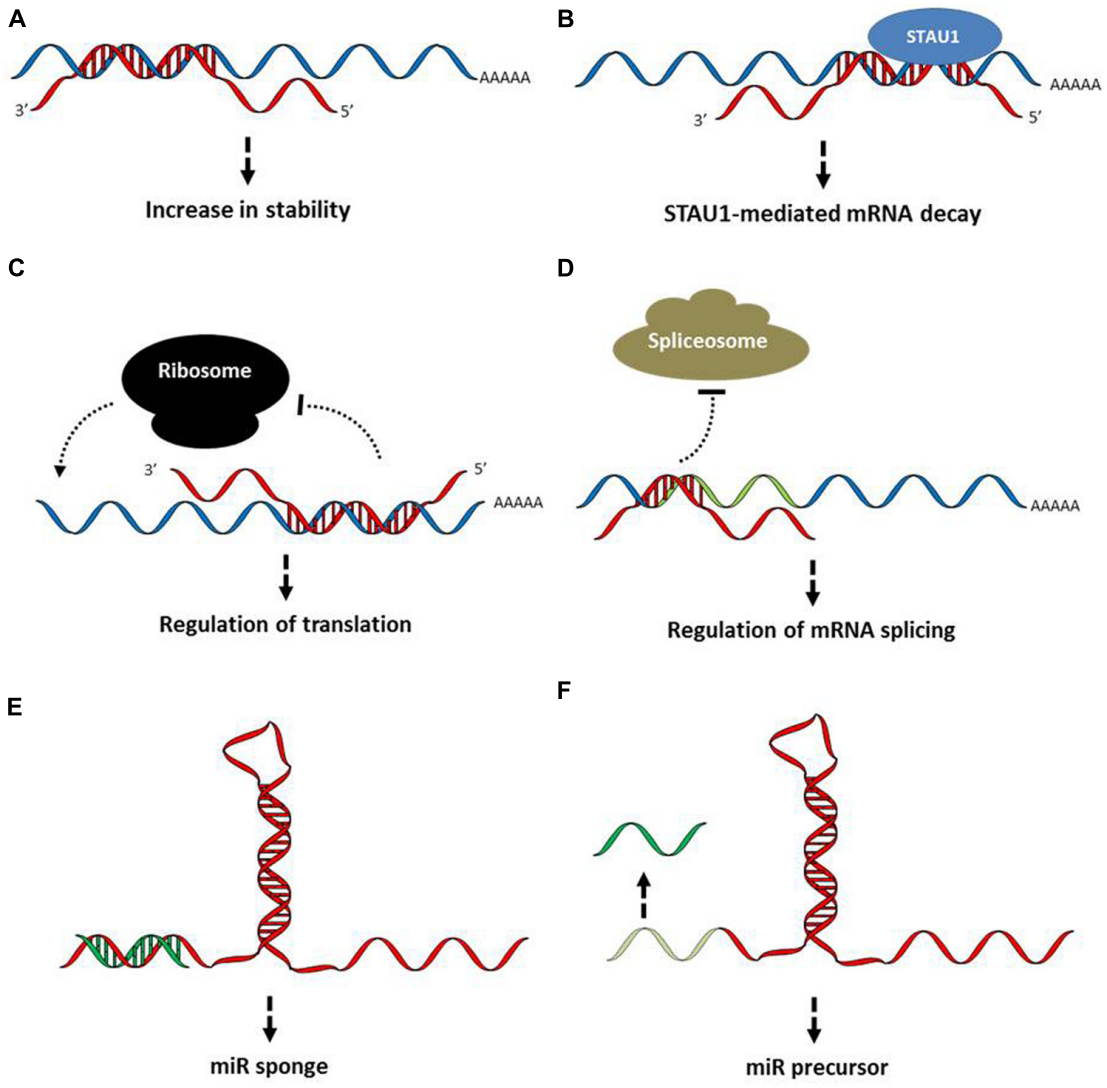
**Functional mechanisms of long non-coding (lncRNA) action at the post-transcriptional levels. (A)** mRNA stabilization. Base pairing between specific regions of a long non-coding antisense RNA and its sense transcript induces stabilization of the target mRNA and increases protein abundance. **(B)** mRNA degradation. Staufen double-stranded RNA-binding protein 1 (STAU1)-mediated mRNA decay is induced when base pairing is formed between the mRNA and a lncRNA. **(C)** Ribosome targeting. Through homologous base pairing with mRNAs and interactions with ribosomal proteins lncRNAs target transcripts to ribosomes or prevent translation. **(D)** Regulation of splicing. Base pairing between mRNAs and lncRNAs may prevent splicing by masking the splicing sites. In addition, lncRNAs are also implicated in the formation and maintenance of nuclear structures involved in alternative splicing of nascent transcripts. **(E)** miR sponge. By sequestering miRs through base pairing formations, lncRNAs affect the expression of the miR target genes. **(F)** Precursor of miRs. LncRNAs can serve as a source of miRs after processing. LncRNAs are shown in red, whereas mRNAs are in blue. See text for examples.

The ability of lncRNAs to recognize complementary sequences allows the regulation of mRNA processing at various steps, including degradation, splicing, translation, or transport (**Figure 1**).

Base pairing between defined regions of the human β-site APP-cleaving enzyme 1 (BACE1) transcript and its antisense lncRNA BACE1-AS induces the mRNA stabilization and consequently the increase in BACE1 protein abundance (Faghihi et al., 2008). Similarly, the lncRNA TINCR (terminal differentiation-induced ncRNA) interacts with a range of differentiation mRNAs including FLG, LOR, ALOXE3, ALOX12B, ABCA12, CASP14, or ELOVL3, to increase their stability (Kretz et al., 2013). In contrast, the recognition of mRNAs by other lncRNAs, such as half-STAU1-binding site RNAs (1/2sbsRNAs) decrease target mRNA stability by inducing STAU1 recruitment and the STAU1-mediated mRNA decay pathway (Gong and Maquat, 2011).

The translational process may also be modulated positively or negatively by lncRNA–mRNA pairing. For example, the antisense lncRNA ULCH-AS1 (ubiquitin carboxy-terminal hydrolase L1 antisense RNA 1) enhances ULCH mRNA translation (Carrieri et al., 2012), whereas lincRNA-p21 or pseudo-NOS suppress target mRNA translation (Korneev et al., 1999; Yoon et al., 2012).

The lncRNA MALAT1 (metastasis associated lung adenocarcinoma transcript 1) regulates pre-mRNA alternative splicing by modulating active serine/arginine splicing factors levels (Tripathi et al., 2010). In this case, the modulation of the mRNA processing is not achieved by a lncRNA–mRNA pairing mechanism but rather by the MALAT1-mediated modulation of the distribution of various splicing factors in nuclear speckle domains. However, antisense transcripts may also affect alternative splicing of their sense transcripts by virtue of masking splice sites by base complementarity (Krystal et al., 1990; Khochbin et al., 1992; Beltran et al., 2008). For example, a specific isoform of the lncRNA NPPA-AS is capable of down-regulating the intron-retained NPPA (atriuretic peptide precursor A) mRNA variant through RNA duplex formation between the sense and antisense transcripts (Annilo et al., 2009).

## LncRNAs and the Connection with the MicroRNA World

Some lncRNAs act on post-transcriptional regulation through the modulation of the microRNA (miR) pathways. MiRs, a large class of small ncRNA, function by annealing to complementary sites in the coding sequences or 3′-untranslated regions (UTRs) of target mRNAs where they favor the recruitment of protein factors that impair translation and/or promote transcript degradation leading to a decrease in protein abundance (Baek et al., 2008; Bartel, 2009). Specifically, one mechanism by which the BACE1-AS lncRNA enhances BACE1 sense mRNA stability could be by masking the binding site for miR-485-5p (Faghihi et al., 2010). Rather than competing for miR-binding sites, a number of lncRNAs contain miR-binding sites in their sequence and therefore act as “sponges” to sequester miRs away from their mRNA targets. The pseudogene PTENP1 previously considered as biologically inactive was found to sequester miRs, consequently affecting their action on target gene regulation (Poliseno et al., 2010). In particular, the 3′-UTR of the PTENP1 lncRNA binds the same set of miRs targeting the tumor suppressor gene PTEN, then reducing the downregulation of this transcript and thus enhancing PTEN protein abundance. A number of other lncRNAs, including KRASP1, linc-MD1, HULC, or linc-ROR were shown to control mRNA activity through a miR sponge mechanism (Poliseno et al., 2010; Wang et al., 2010, 2013; Cesana et al., 2011). These examples illustrate that lncRNAs could counteract miR actions, but lncRNAs can themselves give rise to miRs and thus favor post-translational control by miR pathways as it is the case for the mouse Dlk1–Dio3 cluster or the BIC lncRNA (Eis et al., 2005; Hagan et al., 2009). Within the Dlk1–Dio3 cluster, *Meg3/Gtl2* contains in its last intron the evolutionarily conserved microRNA miR-770 whereas *Meg8* transcripts have the intron-encoded miR-341, miR-1188, and miR-370. Similarly, miR-155 is processed from sequences present in BIC lncRNA that accumulates in lymphoma cells.

## LncRNAs in the Transcriptional Control

**FIGURE 2:**
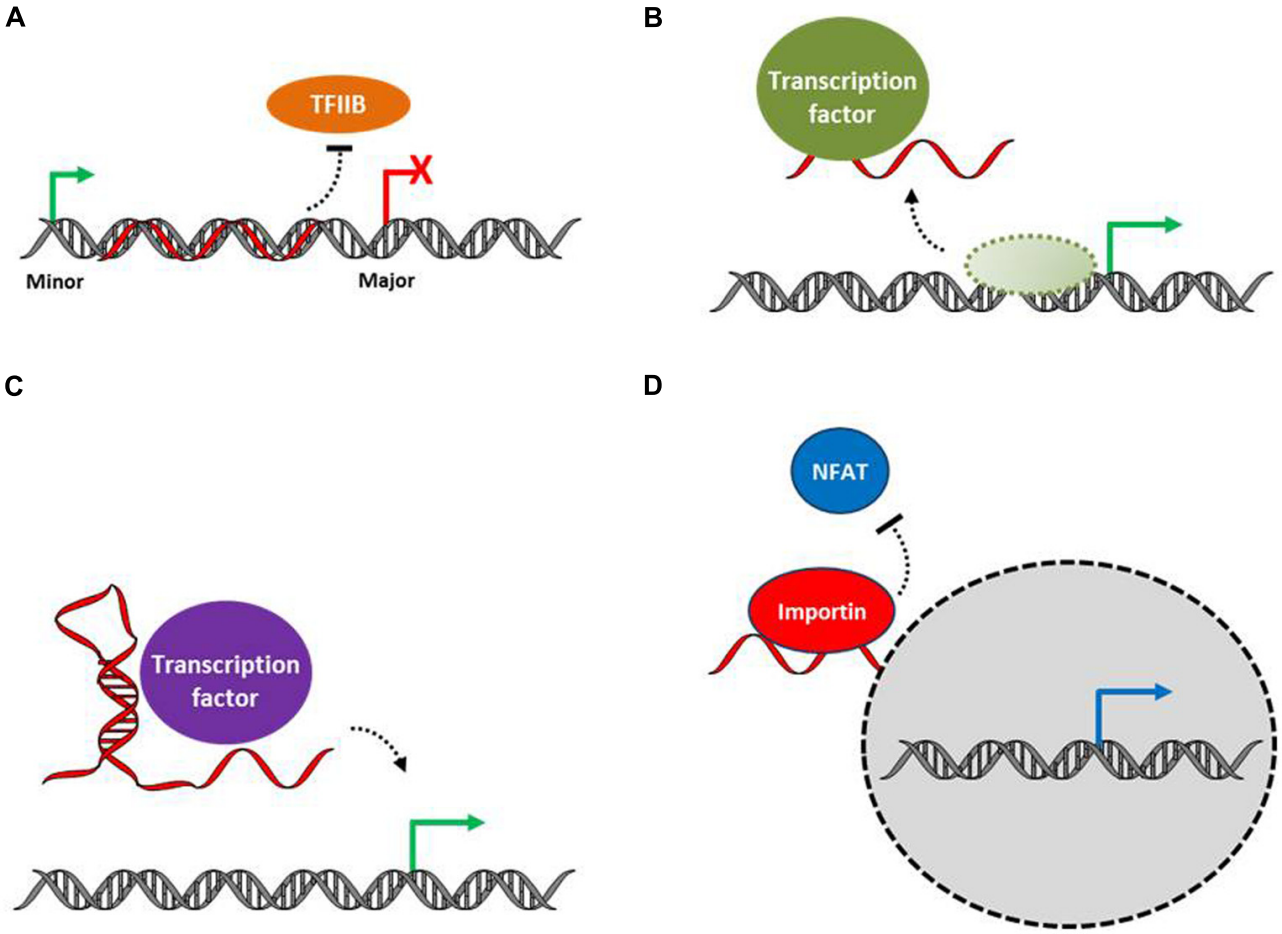
**Functional mechanism of action at the levels of transcriptional regulation. (A)** LncRNA may regulate transcription by virtue of RNA–DNA triplex formation preventing the formation of the transcription initiation complex at promoters. **(B)** LncRNAs can act as decoys by titrating transcription factors away from their cognate promoters. **(C)** LncRNAs can regulate transcription through the targeting of transcription factors to promoters or acting as co-factors involved in transcription factor activity. **(D)** LncRNA can also control transcription factor trafficking. LncRNAs are shown in red.

A number of evidences indicate that lncRNAs can act at the level of transcription either negatively or positively through a variety of molecular mechanisms (**Figure 2**). The dihydrofolate reductase (DHFR) gene contains a major and a minor promoter. The minor promoter gives rise to a lncRNA that forms a stable triplex lncRNA-DNA association at the major DHFR promoter and interacts with the general transcription factor II B (TFIIB) leading to the dissociation of the transcriptional preinitiation complex at this major promoter and then reducing DHFR expression (Martianov et al., 2007).

Other lncRNAs act as decoys to negatively control transcription by titrating transcription factors away from their cognate promoters. The lncRNA PANDAR (promoter of CDKN1A antisense DNA damage activated RNA) is induced in a TP53-dependent manner and inhibits apoptotic gene expression to favor cell-cycle arrest through direct interaction with, and sequestration of NFYA, a transcription factor controlling the apoptotic program upon DNA damage (Hung et al., 2011). Similarly, the lncRNA GAS5 (growth arrest-specific 5) contains an RNA motif derived from a stem-loop structure mimicking a DNA motif corresponding to the glucocorticoid response element. GAS5 binds to the DNA-binding domain of the glucocorticoid receptor, acts as a decoy glucocorticoid response element and is thus competing with DNA sites for binding to the glucocorticoid receptor (Kino et al., 2010).

Rather than acting as molecular decoys, lncRNA could modulate transcription by recruiting factors at target gene promoters or acting as transcription factor co-activators. For example, a lncRNA produced at the 5′ regulatory region of the cyclin D1 (CCND1) gene in response to genotoxic stress tethers and modulates the activity of the RNA-binding protein TLS (translocated in liposarcoma) which in turn inhibits the activity of the histone acetyltransferases CBP (CREB binding protein) and EP300, leading to CCND1 transcriptional repression (Wang et al., 2008). The lncRNA Evf-2 (DLX6-AS1) forms a stable complex with the homeodomain-containing protein DLX2 to induce expression of the adjacent genes at the DLX5/6 locus (Feng et al., 2006). In this later case, the Evf-2 lncRNA functions as a co-factor regulating transcription factor activity.

Other lncRNAs regulate transcription by controlling transcription factor trafficking. As such, the lncRNA NRON (non-protein coding RNA, repressor of NFAT) interacts with importin-beta family members to inhibit nuclear translocation of the inactive dephosphorylated nuclear factor of activated T cells (NFAT) trans-activator (Willingham et al., 2005).

## LncRNAs and Epigenetics

**FIGURE 3:**
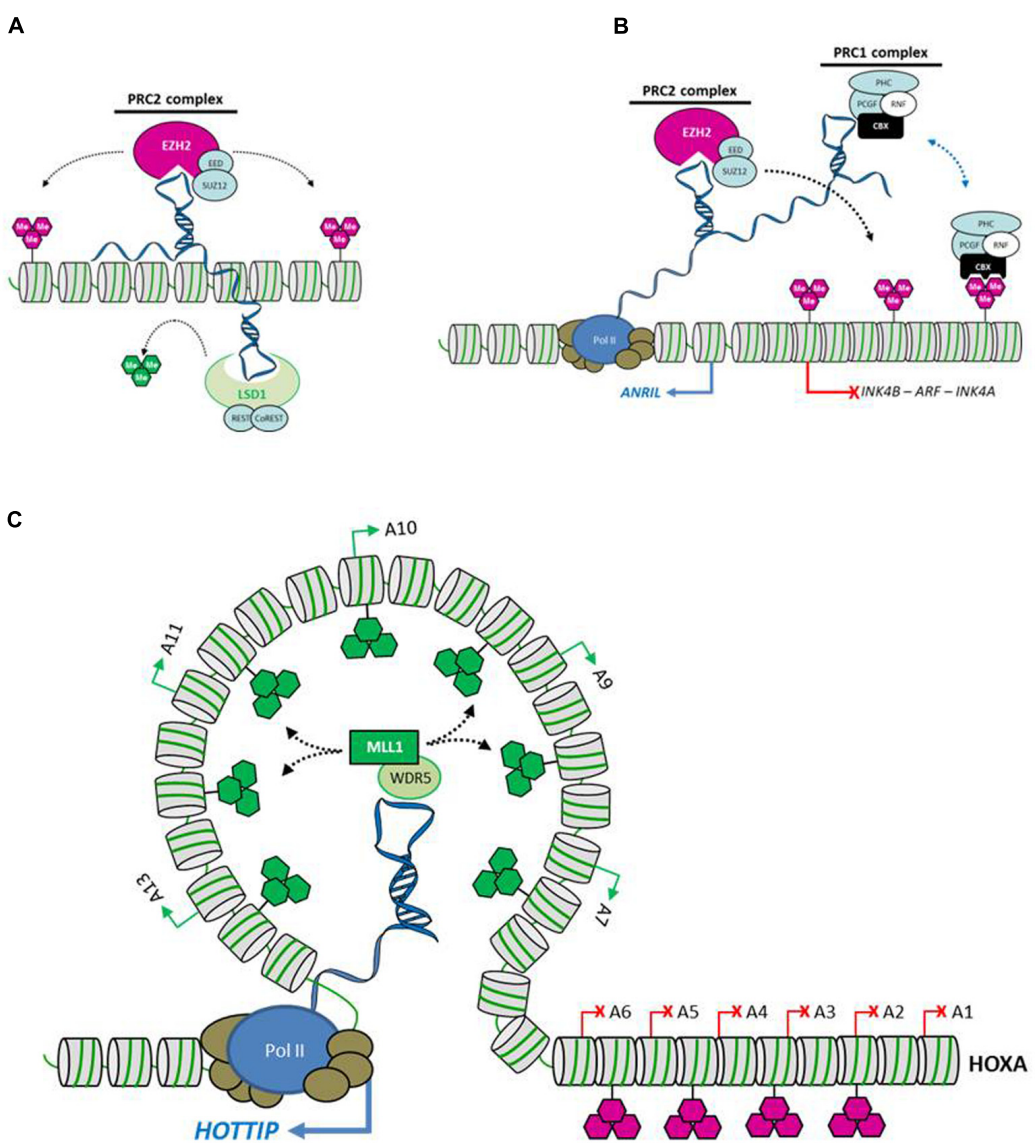
**Examples of lncRNAs controlling chromatin organization. (A)** HOTAIR (HOX transcript antisense RNA) represses transcription in *trans* by recruiting two different chromatin modifying activities. The Polycomb Repressive Complex 2 (PRC2) produces the repressive H3K27me3 marks, whereas the LSD1-CoREST complex is responsible for the removal of the active H3K4me2/3 marks. **(B)** The ANRIL lncRNA represses transcription in *cis* at the INK4B/ARF/INK4A locus by recruiting the Polycomb repressive complexes PRC1 and PRC2. **(C)** The HOTTIP (HOXA transcript at the distal tip) lncRNA activates genes by recruiting the histone modifier complex WDR5-MLL which is responsible for H3K4me3 methylation, and by mediating long-range chromatin looping at one extremity of the HOXA locus. Purple hexagons represent H3K27me3 repressive marks, whereas green hexagons illustrate H3K4me3 activating marks.

LncRNAs have been implicated in the control of gene expression through the recruitment of epigenetic modifiers at specific genomic loci. In eukaryotic chromatin, epigenetic regulation is conveyed by covalent modifications of DNA (methylation, hydroxymethylation), modifications of histone tails (acetylation, methylation, phosphorylation, ubiquitinylation), and the incorporation of various histone variants. These modifications locally change chromatin organization and regulate gene expression without changes in the DNA sequence. A number of evidences indicate that lncRNAs, acting as guides targeting enzymes involved in chromatin modifications, are part of this picture (**Figure 3**).

The lncRNA HOTAIR (HOX transcript antisense RNA) is transcribed from the HOXC locus and targets Polycomb Repressive Complex 2 (PRC2) to silence distantly located genes, including genes at the HOXD locus and 100s of other genes on various chromosomes (Rinn et al., 2007; Zhang et al., 2015). Components of PRC2 trimethylate lysine 27 of histone H3 (H3K27me3) establishing the silent chromatin state (Völkel and Angrand, 2007; Völkel et al., 2015). Interestingly, HOTAIR also binds the LSD1–CoREST complex which possesses a lysine 4 of histone H3 demethylase activity, thus removing an active H3K4me2 chromatin mark (Tsai et al., 2010). Furthermore, deletion analysis of HOTAIR revealed that distinct parts of the lncRNA interact with PRC2 and LSD1 indicating that HOTAIR is able to bridge two independent chromatin modifying activities at a target locus. Indeed, the knockdown of HOTAIR is responsible for the concomitant loss of occupancy of PRC2 and LSD1, and concurrent loss of H3K27me3 and gain of H3K4me2 at target loci. Then, HOTAIR acts as an RNA scaffold targeting two different histone modification activities involved in heterochromatin formation.

The interplay between one lncRNA and different chromatin modifying complexes is also found at the INK4A tumor-suppressor locus. The antisense lncRNA ANRIL (antisense non-coding RNA in the INK4 locus, CDKN2B-AS) which is produced by the INK4B/ARF/INK4A locus binds specifically two Polycomb proteins, CBX7 (PRC1) and SUZ12 (PRC2). Disruption of interaction with both PRC1 and PRC2 proteins impacts the transcriptional repression at the INK4B locus in *cis* (Yap et al., 2010; Kotake et al., 2011). As another example, the lncRNA KCNQ1OT1 (KCNQ1 opposite strand/antisense transcript 1) mediates bidirectional silencing by interacting with chromatin and recruiting the PRC2 complex, as well as the histone methyltransferase G9a (EHMT2), resulting in an increase in the repressive histone modifications H3K27me3 and H3K9me3 at the KCNQ1 domain (Pandey et al., 2008). Thus, similar to HOTAIR and ANRIL, KCNQ1OT1 represents a prototype of a scaffold RNA recruiting multiple sets of chromatin modifying activities involved in target gene silencing. Approximately 20% of lncRNAs, including HOTAIR, ANRIL, KCNQ1OT1, but also XIST, RepA, HEIH, PCAT-1, H19, or linc-UBC1 (Zhao et al., 2008; Maenner et al., 2010; Prensner et al., 2011; Yang et al., 2011; Luo et al., 2013; He et al., 2013), are believed to guide PRC2 activity to target genes, indicating that lncRNA-mediated targeting of PRC2 at chromatin is a widely used strategy to repress gene expression through a chromatin reorganization mechanism (Khalil et al., 2009).

In contrast, the lncRNA HOTTIP (HOXA transcript at the distal tip) mediates transcriptional activation by controlling chromatin modification and organization (Wang et al., 2011). HOTTIP is produced from the 5′-end of the HOXA locus, downstream of HOXA13. The knockdown of HOTTIP decreases expression of HOXA genes in *cis*, with an efficacy that correlates with the proximity of the HOXA genes relative to the HOTTIP transcriptional unit. At the target genes, knockdown of HOTTIP results in the loss of activating H3K4me3 and H3K4me2 epigenetic marks, together with the decreases in occupancy of the MLL1 protein complex responsible for the establishment of these histone modifications. Furthermore, chromosome conformation capture carbon copy (C5) assays revealed abundant long-range looping interactions, bridging the transcribed target HOXA genes into proximity of the HOTTIP transcriptional unit. Thus, the mechanism by which the lncRNA HOTTIP controls HOXA expression relies on its potential to guide the histone methyltransferase MLL1 at target HOXA gene promoters, and on the formation of chromatin loops that connect distantly expressed HOXA genes to HOTTIP transcripts.

A role of lncRNAs in chromatin loop formation has also been described for the lncRNA CCAT1-L (Xiang et al., 2014). Indeed, CCAT1-L, is transcribed from a locus upstream of MYC and plays a role in MYC transcriptional regulation by promoting long-range chromatin looping.

Thus, lncRNAs, through the recruitment of chromatin modifiers and/or the induction of chromatin loops will modulate the chromatin conformation and will format the genome in a particular configuration. This lncRNA-mediated genome formatting emerges as a crucial and fundamental mechanism by which lncRNA may act on gene expression programs.

## H19, a Prototype of a Multitask lncRNA

As discussed above, lncRNAs can regulate genome expression through different molecular mechanisms. However, several lncRNAs use multiple strategies that, in combination, may be required for their biological function. The action of the lncRNA H19 on gene expression illustrates the complexity of the combinatorial mechanisms of regulation achieved by a single lncRNA. H19 was the first lncRNA discovered (Brannan et al., 1990). Furthermore, H19 and its neighboring IGF2 gene located at position 11p15.5 are subjected to genomic imprinting and the study of the gene regulation at this locus serves as a model for understanding the molecular mechanisms involved in this genomic regulation. In addition, alterations of gene expression at the H19/IGF2 locus are associated to malignancies and developmental disorders. Loss of heterozygosity including loss of imprinting could be responsible for a loss of expression or a biallelic expression of these genes. Patients suffering from Beckwith–Wiedemann syndrome (BWS, OMIM 130650; Choufani et al., 2010) exhibit a loss of H19 expression and a biallelic expression of IGF2. BWS is associated with fetal and postnatal overgrowth and increased risk of embryonic or childhood cancers such as Wilm’s tumors. Loss of IGF2 expression with a biallelic H19 expression is responsible for 20 to 60% of cases of Silver–Russel syndrome (SRS, OMIM 180860; Penaherrera et al., 2010). SRS is an intrauterine growth delay associated to an altered postnatal growth with facial dysmorphia and corporal asymmetry. Numerous studies including ours indicate that H19 may play a key role in tumorigenesis and could contribute to tumor progression and aggressiveness. H19 overexpression has also been reported in various cancer tissues including breast (Adriaenssens et al., 1998; Lottin et al., 2002), bladder (Cooper et al., 1996), lung (Kondo et al., 1995), and esophageal cancers (Hibi et al., 1996). Several lines of evidence indicate that H19 could play a role in tumor invasion and angiogenesis. In breast cancer, the oncogenic role of H19 has been well established (Berteaux et al., 2005), even if the precise molecular mechanisms involved in tumorigenesis are not yet fully understood.

**FIGURE 4:**
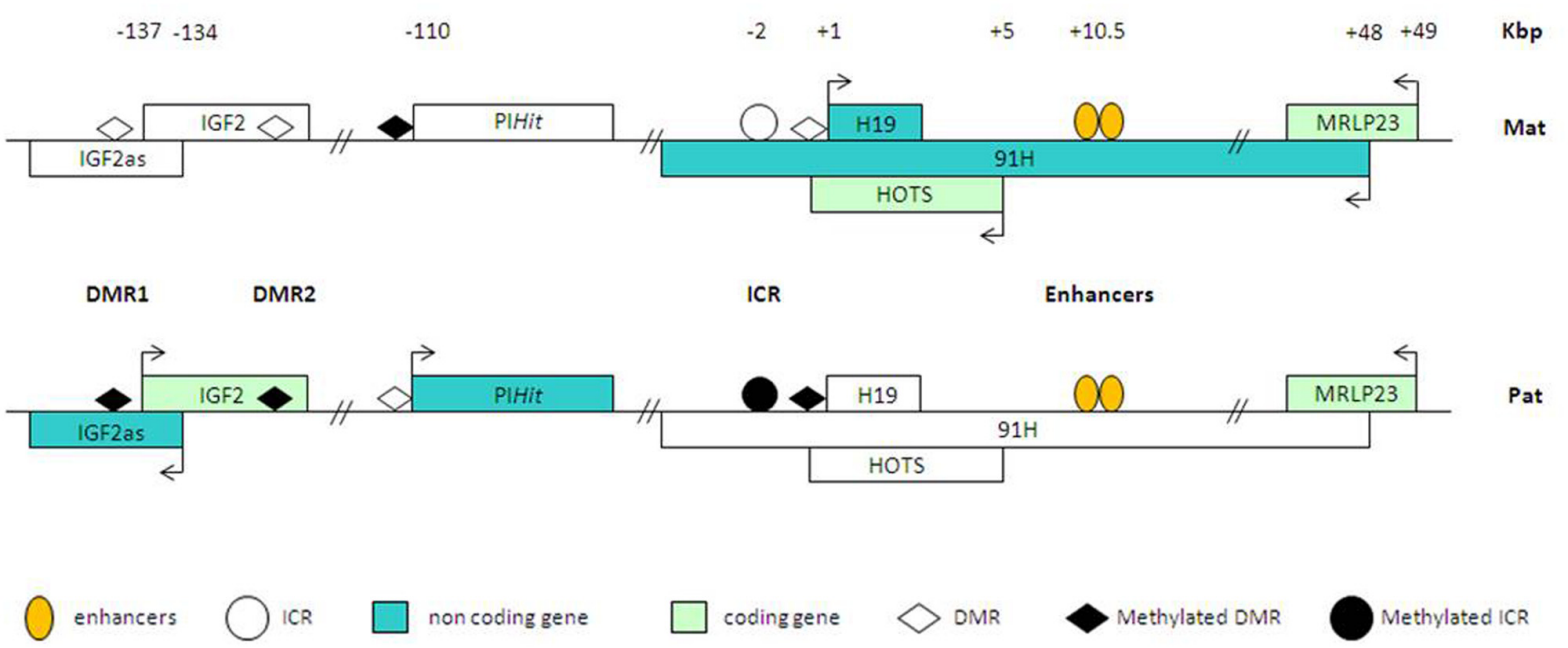
**Schematic representation of the transcriptional complexity at the H19/IGF2 locus.** Non-coding transcripts at the H19/IGF2 are shown as blue squares when they are expressed. Coding genes are in green, when expressed. The differences in gene expression between the paternal and maternal alleles are shown. The DNA methylation status of the regulatory elements ICR (imprinting control region) and DMRs (differentially methylated regions) is indicated for the paternal and maternal alleles.

At the H19/IGF2 locus, both genes share a common set of enhancers located downstream of the H19 gene (**Figure 4**). The ICR (imprinting control region), located 2 kbp upstream of the H19 promoter, controls the monoallelic expression of H19 and IGF2 by insulating communication between the 3′ enhancers and the IGF2 promoter. The chromatin insulator property of the H19/IGF2 ICR is regulated by the insulator CTCF (CCTC-binding factor), which binds specifically to the unmethylated maternal allele. On the paternal allele, the ICR methylation does not allow CTCF binding and leads to IGF2 expression (reviewed in Lewis and Murrell, 2004). The H19/IGF2 locus contains other differentially methylated regions (DMRs), with DMR1 being a methylation-sensitive silencer and DMR2 being a methylation-sensitive activator (Constancia et al., 2000; Murrell et al., 2004). CTCF binding to the maternal ICR regulates its interaction with matrix attachment region 3 (MAR3) and DMR1 at IGF2, thus forming a tight loop around the maternal IGF2 locus which may contribute to its silencing. These interactions restrict the physical access of distal enhancers to the IGF2 promoter (Weber et al., 2003; Murrell et al., 2004; Kurukuti et al., 2006). Furthermore, several lncRNAs are produced at the H19/IGF2 locus adding further complexity to the locus regulation. The first antisense transcript at the H19/IGF2 locus is the lncRNA IGF2-AS (3–4 kb) discovered in 1991 in chicken (Rivkin et al., 1993; Moore et al., 1997). IGF2-AS and IGF2 are coregulated at the transcriptional levels but the function of this IGF2-AS lncRNA remains unclear. The lncRNA 91H (about 120 kb) is transcribed from the maternal allele (Berteaux et al., 2008). Recently, at the same position, a new protein coding gene HOTS (6 kbp) has been described (Onyango and Feinberg, 2011) but the relationship between the HOTS and 91H is still not clear. However, these two transcripts are transcribed in an antisense orientation compared to H19. An additional lncRNA produced by the H19/IGF2 locus has been identified (Court et al., 2011). This PIHit (paternally expressed IGF2/H19 intergenic transcript) lncRNA is a 5–6 kb transcript expressed from the paternal allele after birth. Thus, the genomic organization of coding and non-coding transcripts illustrates the complexity of the interleaved networks of lncRNAs expressed from the H19/IGF2 locus.

**FIGURE 5:**
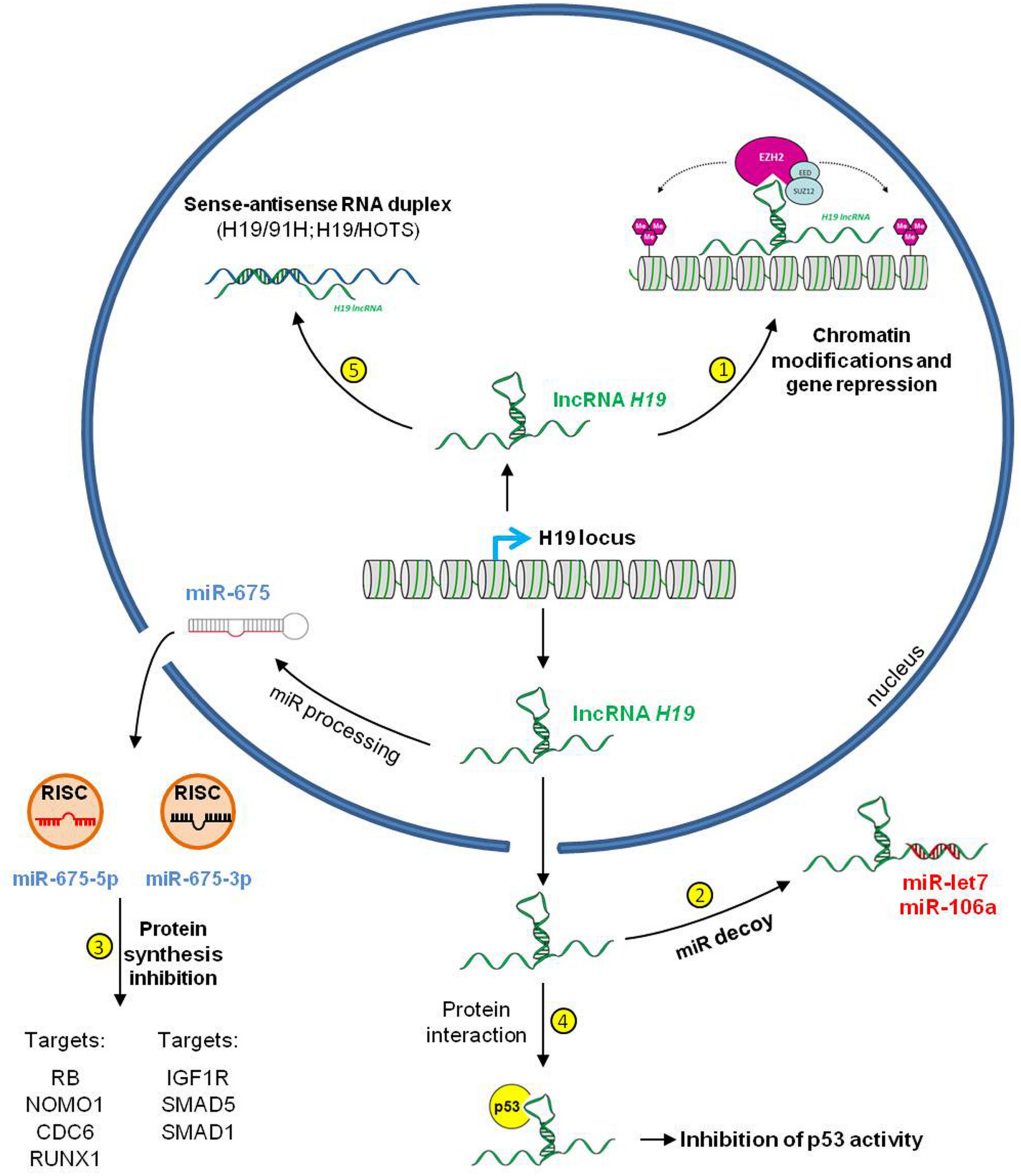
**The multifaceted action of the lncRNA H19.** The lncRNA H19 controls genome expression at multiple levels. H19 acts on chromatin organization through the recruitment of chromatin modifying complex PRC2 **(1)** and on post-transcriptional control as a miR decoys sequestering miR-106a and miR-let7 **(2)** or as a precursor for miR-675-5p and miR-675-3p **(3)** H19 also interact with p53 (TP53) and inactivate the tumor suppressor protein action **(4)** Furthermore, possible base pairing between H19 and the antisense transcripts 91H and HOTS may have biological outcomes **(5)**.

To complicate matters further, H19 lncRNA mechanisms of action appear to be extremely diverse, acting at various levels (**Figure 5**). H19 has been shown to guide chromatin modifying enzymes to specific loci. In particular, Luo et al. (2013) have shown that H19 binds to and recruit the histone methyltransferase EZH2 at the E-cadherin promoter, leading to an increase in H3K27me3 repressive marks and to the silencing of the E-cadherin gene in bladder cancer. PRC2 protein members are not the only chromatin modifying factors interacting with H19 since it has been shown that this lncRNA physically binds to the methyl-CpG-binding domain protein 1 (MBD1). The H19-MBD1 complex is then recruited at several imprinted genes including IGF2, SLC38A4, and PEG1 (Monnier et al., 2013). This recruitment also induces methylation at lysine 9 of histone H3 (H3K9me3), probably via the additional interaction with an H3K9 histone methylransferase.

The multifaceted action of H19 is also illustrated by its dual interaction with miR pathways. On one hand, the lncRNA H19 acts as miR sponge to sequester miR-106a as well as the mir-let7 family members (Kallen et al., 2013; Imig et al., 2015). On the other end, H19 serves as a precursor of miR-675 that will in turn, post-translationally regulate a number of targets involved in cell tumorigenicity, including RB, IGFR1, SMAD1, SMAD5, CDC6, NOMO1, or RUNX1 (Cai and Cullen, 2007; Tsang et al., 2010; Gao et al., 2012; Keniry et al., 2012; Dey et al., 2014; Zhuang et al., 2014). The role of H19 in tumor progression could also be mediated through its interaction with the tumor-suppressor TP53 protein. This association results in partial TP53 inactivation (Yang et al., 2012).

Several evidences also indicate that the H19 lncRNA controls IGF2 expression at the translational and/or post-translational levels (Li et al., 1998), suggesting that other mechanisms by which H19 exerts its action remain to be deciphered. Similarly, the possible role of RNA duplex formation between H19 and the antisense transcripts 91H and HOTS requires investigations.

## LncRNAs in Human Diseases

Given the wide range of molecular actions achieved by the lncRNAs and their roles in various physiological processes, it is not surprising that they have been shown to be involved in many human diseases. A number of data indicate that alterations of lncRNA expression lead to tumorigenesis through changes at the chromatin, transcriptional or post-transcriptional levels that impact target genes expression (**Table 1**). Since lncRNAs are regulating a different cellular pathways, growing evidences suggest that they could play a role in a large number of other human disorders including metabolic diseases, neurodegenerative and psychiatric disorders, cardiovascular and immune dysfunctions (Taft et al., 2010; Esteller, 2011; Harries, 2012; Shi et al., 2013; Clark and Blackshaw, 2014).

**Table 1:** Examples of long non-coding RNAs (lncRNAs) associated with human disorders.

lncRNA	Cancer/disease	Mechanisms of action	Reference
ANRIL	Neurofibromatosis type 1, prostate cancer, melanoma, acute lymphoblastic leukemia	Chromatin modification via the recruitment of the Polycomb Repressive Complex 2 (PRC2) at the INKB/ARF/INK4A tumor suppressor locus	Pasmant et al. (2007), Pasmant et al. (2011), Yap et al. (2010), Iacobucci et al. (2011)
HOTAIR	Hepatocellular carcinoma, colorectal cancer, breast cancer, glioblastomas	Chromatin modification via the recruitment of PRC2 and LSD1 in *trans*.	Gupta et al. (2010), Kogo et al. (2011), Yang et al. (2011), Zhang et al. (2015)
H19	Colorectal, gastric, breast, lung, esophageal, bladder, pancreas, ovary cancers	Chromatin modification via the recruitment of PRC2; Decoy for miR-Let-7; source of miR-675; TP53 inactivation	Kondo et al. (1995), Cooper et al. (1996), Hibi et al. (1996), Lottin et al. (2002), Berteaux et al. (2005), Tsang et al. (2010), Yang et al. (2012), Luo et al. (2013), Ma et al. (2014), Zhuang et al. (2014)
HEIH	Hepatocellular carcinoma	Chromatin modification via the recruitment of PRC2	Yang et al. (2011)
PCAT-1	Prostate cancer	Chromatin modification via the recruitment of PRC2	Prensner et al. (2011)
linc-UBC1	Bladder cancer	Chromatin modification via the recruitment of PRC2	He et al. (2013)
BACE1-AS	Alzheimer’s disease	Increase in mRNA stability	Faghihi et al. (2008)
GAS5	Breast, bladder cancers	Decoy for the glucocorticoid receptor; regulation of CDK6 expression	Mourtada-Maarabouni et al. (2009), Kino et al. (2010), Liu et al. (2013)
PTENP1	Prostate cancer	miR decoy	Poliseno et al. (2010)
KCNA2-AS	Neuropathic pain	Decrease of KCNA2 expression	Zhao et al. (2013)
MIAT	Schizophrenia	Component of the nuclear matrix involved in mRNA splicing	Barry et al. (2014), Ishizuka et al. (2014)
MALAT1	Lung cancer	Alternative splicing regulation	Schmidt et al. (2011)

## Perspectives and Concluding Remarks

LncRNAs represent a large part of the transcriptome and a very heterogeneous class of transcripts in terms of genomic organization and modes of action. Many of them are considered as key regulators of gene expression and thus, lncRNAs constitute an additional layer controlling the cellular programs. LncRNAs regulate diverse expression steps at the levels of chromatin rearrangement, transcriptional control, and/or post-transcriptional processing. By these actions, lncRNAs are involved in numerous physiological functions and in many cases lncRNA alterations are associated with human disorders.

The fact that lncRNAs can be deregulated in tumors and other human pathologies, make them attractive candidates as biomarkers and as targets for therapy. LncRNAs may be down-regulated at the RNA levels by targeting their sequence. As so, short interfering RNAs (siRNAs) designed to perfectly match exact stretches of nucleotides, guarantee a high degree of specificity leading to lncRNA degradation. The power of the siRNA approach is illustrated by the success of a number of preclinical studies where siRNAs targeted mRNAs (Kaur et al., 2014). Similar approaches can thus be envisioned to target non-coding RNAs. Indeed, siRNAs have also been used to target miRs, leading to heart regeneration in an *in vivo* mouse model (Aguirre et al., 2014) and the use of siRNAs has been proposed in a therapeutic strategy targeting the lncRNA HOTAIR in endometrial carcinoma (Huang et al., 2014). Similarly, antisense oligonucleotides, single-strand DNA, or RNA molecules of 8 to 50 nucleotides can be used to target lncRNA. Specifically, *in vivo* and *in vitro* experiments revealed that antisense oligonucleotides directed against the lncRNA MALAT1 inhibit its expression and drastically reduce lung cancer metastasis (Gutschner et al., 2013; Tripathi et al., 2013).

In this context, further exploration in the complexity of the lncRNA world promises the emergence of novel therapeutic opportunities.

## Conflict of Interest Statement

The authors declare that the research was conducted in the absence of any commercial or financial relationships that could be construed as a potential conflict of interest.
